# Effectiveness of the HEAR-Aware App for Adults Not Ready for Hearing Aids, but Open to Self-Management Support: Results of a Randomized Controlled Trial

**DOI:** 10.1097/AUD.0000000000001533

**Published:** 2024-06-04

**Authors:** Vanessa Feenstra-Kikken, Sjors Van de Ven, Birgit I. Lissenberg-Witte, Marieke Pronk, Cas Smits, Barbra H. B. Timmer, C. Polleunis, Jana Besser, Sophia E. Kramer

**Affiliations:** 1 Department of Otolaryngology—Head and Neck Surgery, Amsterdam University Medical Center, Vrije Universiteit Amsterdam, Ear & Hearing, Amsterdam Public Health Research Institute, Amsterdam, the Netherlands; 2 Amsterdam University Medical Center Location Vrije Universiteit Amsterdam, Department of Epidemiology and Data Science, Amsterdam, the Netherlands; 3 Amsterdam University Medical Center Location Universiteit van Amsterdam, Department of Otolaryngology—Head and Neck Surgery, Ear & Hearing, Amsterdam Public Health Research Institute, Amsterdam, the Netherlands; 4 School of Health and Rehabilitation Sciences, The University of Queensland, Brisbane, Australia; 5 Science and Technology, Sonova Aktien-gesellschaft, Stäfa, Switzerland; 6 Schoonenberg HoorSupport, Rotterdam, the Netherlands.

**Keywords:** Adults, Ecological momentary assessment, Effectiveness, HEAR-aware, Hearing loss, Readiness for action, Self-management, Smartphone app, Stages of change

## Abstract

**Introduction:**

Recently, the HEAR-aware app was developed to support adults who are eligible for hearing aids (HAs) but not yet ready to use them. The app serves as a self-management tool, offering assistance for a range of target behaviors (TBs), such as communication strategies and emotional coping. Using ecological momentary assessment and intervention, the app prompts users to complete brief surveys regarding challenging listening situations they encounter in their daily lives (ecological momentary assessment). In response, users receive educational content in the form of “snippets” (videos, texts, web links) on the TBs, some of which are customized based on the reported acoustic environmental characteristics (ecological momentary intervention). The primary objective of this study was to assess the effectiveness of the HEAR-aware app in enhancing readiness to take action on various TBs and evaluate its impact on secondary outcomes. The secondary objective was to examine the app’s usability, usefulness, and user satisfaction.

**Methods:**

A randomized controlled trial design with two arms was used. Participants with hearing loss aged 50 years and over were recruited via an HA retailer and randomly assigned to the intervention group (n = 42, mean age = 65 years [SD = 9.1]) or the control group (n = 45, mean age = 68 years [SD 8.7]). The intervention group used the app during 4 weeks. The control group received no intervention. All participants completed online questionnaires at baseline (T0), after 4 weeks (T1), and again 4 weeks later (T2). Participants’ readiness to take action on five TBs was measured with The Line Composite. A list of secondary outcomes was used. Intention-to-treat analyses were performed using Linear Mixed effect Models including group (intervention/control), time (T0/T1/T2), and Group × Time Interactions. In addition, a per protocol analysis was carried out to explore whether effects depended on app usage. For the secondary aim the System Usability Scale (SUS), the Intrinsic Motivation Inventory, item 4 of the International Outcome Inventory-Alternative Intervention (IOI-AI), and a recommendation item were used (intervention group only at T1).

**Results:**

For objective 1, there was no significant group difference for The Line Composite over the course of T0, T1, and T2. However, a significant (*p* = 0.033) Group × Time Interaction was found for The Line Emotional coping, with higher increase in readiness to take action on emotional coping in the intervention group than in the control group. The intention-to-treat analyses revealed no other significant group differences, but the per protocol analyses showed that participants in the intervention group were significantly more ready to take up Assistive Listening Devices (The Line Assistive Listening Devices) and less ready to take up HAs (Staging Algorithm HAs) than the control group (*p* = 0.049). Results for objective 2 showed that on average, participants rated the app as moderately useful (mean Intrinsic Motivation Inventory score 5 out of 7) and its usability as “marginal” (mean SUS score 68 out of 100) with about half of the participants rating the app as “good” (SUS score >70) and a minority rating is as “unacceptable” (SUS score ≤50).

**Conclusions:**

This study underscores the potential of self-management support tools like the HEAR-aware app in the rehabilitation of adults with hearing loss who are not yet ready for HAs. The range in usability scores suggest that it may not be a suitable intervention for everyone.

## INTRODUCTION

Acquired hearing impairment is one of the most prevalent chronic conditions and when unaddressed the third leading cause of disability worldwide (Global Burden of Disease 2016; World Health Organization [WHO] 2021). Daily life activities and roles that rely on spoken communication are particularly affected by reduced hearing ability.

Hearing impairment is strongly age-related and usually becomes evident around the age of 50 (Gates & Mills 2005). Hearing aids (HAs) are still the standard rehabilitation option (Boothroyd 2007; Ferguson et al. 2017). However, the majority of adults with hearing problems postpone help-seeking and rehabilitation by 7 to 10 years (Davis et al. 2007), or do not seek help at all (Knudsen et al. 2010). Around 60 percent of the adults aged 50+ years with hearing impairment who might benefit from HAs do not own them (Hartley et al. 2010). Key reasons for low HA uptake are: low self-reported hearing disability (partly stemming from low hearing loss awareness and/or acceptance), high stigma attached to hearing impairment and HAs, low social support to get HAs, limited benefits of HAs and financial costs (Knudsen et al. 2010; Pronk et al. 2017; Franks & Timmer 2023).

Rehabilitation options for adults with hearing impairment who are not ready for an HA are limited. Very few health care providers offer programs targeting self-management of hearing problems (Laplante-Lévesque et al. 2010). Self-management is the active and ongoing process of acquisition, mastery, and application of an array of skills and knowledge needed to manage the multidimensional impact of hearing impairment (National Health Priority Action Council 2006). A study by Laplante-Lévesque et al. (2011) indicated that a substantial number of adults with hearing impairment may prefer support programs over HAs: 24% of their participants seeking hearing help care for the first time preferred a self-management program over HAs. Interventions focusing on self-management of hearing problems have proven to be effective in training communication strategies, enhancing hearing loss acceptance, increasing hearing-related knowledge, self-efficacy, and involvement of significant others (Kramer et al. 2005; Öberg et al. 2014; Thorén et al. 2014; Ferguson et al. 2016; Preminger & Rothpletz 2016; Öberg 2017; Hickson et al. 2019; Gomez & Ferguson 2020; Meijerink et al. 2020). Nonetheless, most of these interventions were designed with fixed content for all participants, offered via interactive in-person meetings in group format (Hickson et al. 2007; Preminger & Rothpletz 2016) or individually (Hickson et al. 2019) or over the Internet (Thorén et al. 2014; Ferguson et al. 2016; Öberg 2017; Meijerink et al. 2020) with limited options for automated tailoring of the program to an individual’s particular needs or circumstances. With the exception of the (Individualized) Active Communication Program (I-)ACE (Hickson et al. 2007, 2019; Thorén et al. 2014), these are not offered independently of HAs. Also, most existing self-management programs are comprehensive requiring multiple (virtual) visits, possibly contributing to low adherence (Laplante-Lévesque et al. 2010; Meijerink et al. 2020). Lastly, to our knowledge, current interventions elicit awareness of hearing limitations mainly via human interaction that is secured within the program (i.e., via peer-to-peer interaction or active involvement of significant others). Eliciting awareness is generally viewed as important because a lack of it is considered a key barrier to taking steps in the hearing help-seeking journey (Knudsen et al. 2010; Pronk et al. 2017, 2020; Timmer et al. 2021). By actively talking about one’s hearing problems and comparing one’s own views and observations with those of others, and practicing communication strategies together, may bring about such awareness. To our knowledge, facilitating awareness through observing oneself real-time, in one’s own environment, has not been examined before.

The present study is part of the larger HEAR-aware project (Pronk et al. 2020, 2024) which was initiated to design and evaluate a smartphone application (app) aimed at self-management of hearing problems. It was hypothesized that intervention uptake and self-management among adults with hearing loss would be promoted when small, accessible, stand-alone pieces of educational content would be offered in a timely and situationally meaningful manner. Such delivery would be facilitated by answering questions about the (difficult) listening situations individuals encountered in daily life, that is, via ecological momentary assessment (EMA) (Pronk et al. 2020). Such an approach would encourage someone subtly to think about their hearing abilities. EMA holds unique potential to capture real-time experiences in an individuals’ natural environment (Shiffman et al. 2008; Timmer et al. 2018; Holube et al. 2020) and to receive interventional content in real-time extending EMA to ecological momentary intervention (EMI) (McDevitt-Murphy et al. 2018). Smartphone apps are considered particularly suitable for EMA and EMI (Holube et al. 2020; Pronk et al. 2020; Timmer et al. 2021). Apps in the hearing rehabilitation domain have shown to have great potential to improve intervention delivery and to reach large numbers of target groups against relatively low costs (Ozdalga et al. 2012; Saunders & Chisolm 2015; Paglialonga et al. 2018).

In light of the above, the HEAR-aware app was developed. The rationale, theoretical underpinnings, and initial set-up of the app are described in Pronk et al. (2020). Briefly, the HEAR-aware app integrates EMA and EMI techniques and facilitates delivery of small pieces of stand-alone, educational information partly tailored to the acoustical and situational environment of the user (Pronk et al. 2020). The HEAR-aware app’s ultimate aim is to promote individuals’ sense of self-management. Promotion of self-management is operationalized as: (1) increased readiness to take action on five target behaviors (TBs), namely applying communication strategies, improving emotional coping, seeking social support, taking up HAs, and taking up assistive listening devices (ALDs) and (2) improved generic hearing loss self-management (covering knowledge, symptoms monitoring management, emotional management). Acceptability (usage, adherence, usability, usefulness, and satisfaction) of the first prototype was tested by Pronk et al. (2024).

Low readiness to take action on hearing problems has been found to be a key barrier to hearing intervention uptake (Laplante-Lévesque et al. 2012, 2013; Saunders et al. 2016; Pronk et al. 2017). HEAR-aware draws on the idea that readiness can be different for different TBs. Because traditional readiness measures are generic in nature and do not explicitly specify what “taking action on hearing” means, TB-specific readiness measures (Pronk et al. 2020, 2024) were used in the present study and considered the main outcomes of interest.

The aim of this study was two-fold. Objective 1 was to evaluate the effectiveness of the HEAR-aware app through a randomized controlled trial. The primary outcome measure was participants’ TB-specific readiness to take action on their hearing problems as measured with The Line Composite (see Materials and methods). We hypothesized that HEAR-aware app use would lead to an increase in readiness to take action on hearing loss. Objective 2 of this study was to evaluate the app’s overall usability, usefulness, and user satisfaction in the participants allocated to the intervention group.

## MATERIALS AND METHODS

### Study Design

This study had a randomized controlled trial (RCT) design with two arms. Participants were randomly assigned (1:1) to the intervention (HEAR-aware app) or to the control group. Both groups completed three sets of online questionnaires via the program Castor EDC (2023). The first set was completed at baseline, which was before the intervention or control period but after randomization (T0). After completing the T0 questionnaires, the intervention group was provided access to the app during a period of 4 weeks. The control group did not receive any intervention during these 4 weeks. The next set of questionnaires was completed immediately after this intervention or control period (T1). The last set of questionnaires (T2) was completed 4 weeks later. At enrollment, control participants were informed that they would have access to the app after completion of the study (i.e., when the app period was finished for all participants in the intervention group and the particular control participant had completed the T2 questionnaires). The design of the study has been described and listed in the ISRCTN registry with study ID ISRCTN93742150. Ethical approval of the study was obtained from the Ethical Committee of Amsterdam UMC, location VUmc. We closely adhered to the CONSORT 2010 checklist of information to include when reporting an RCT study (Schulz et al. 2010).

### Intervention

The goal of the HEAR-aware app was to help adults with hearing loss self-manage their hearing problems. The app provides educational content (snippets) tailored to a person’s acoustical environment and associated listening challenges (the EMI element of the app, see further later). Participants in the intervention group were sent an email containing a manual with detailed, step-by-step instructions on how to download the app onto their smartphones. They were requested to confirm the successful download by replying to the e-mail. Successful app start-up was also monitored via an online content management system (CMS). In cases where participants faced difficulties during the download process, they were provided with one-on-one instructions via telephone support by a member of the research team or through e-mail, depending on their preference. Once the app was installed on the participant’s smartphone, three times a day (at 9.30 A.M., 1 P.M., and 7:30 P.M.), a push-notification was shown inviting users for a short multiple-choice survey to add and reflect on future, current, or recently passed listening situations that were difficult to manage for them because of their hearing impairment (the EMA element of the app). Participants could select one of 15 predefined listening situations (e.g., 1-on-1 conversation, small/big group conversation, watching TV). Push-notifications were non-compulsory, they could be discarded or temporarily turned off by the user. In addition to the invitations via push-notifications, users could also self-initiate a survey and thus report on new listening situations at any time during the day. Examples of screenshots of the HEAR-aware app are provided in Figure 1 in Supplemental Digital Content, http://links.lww.com/EANDH/B424.

**Fig. 1.**
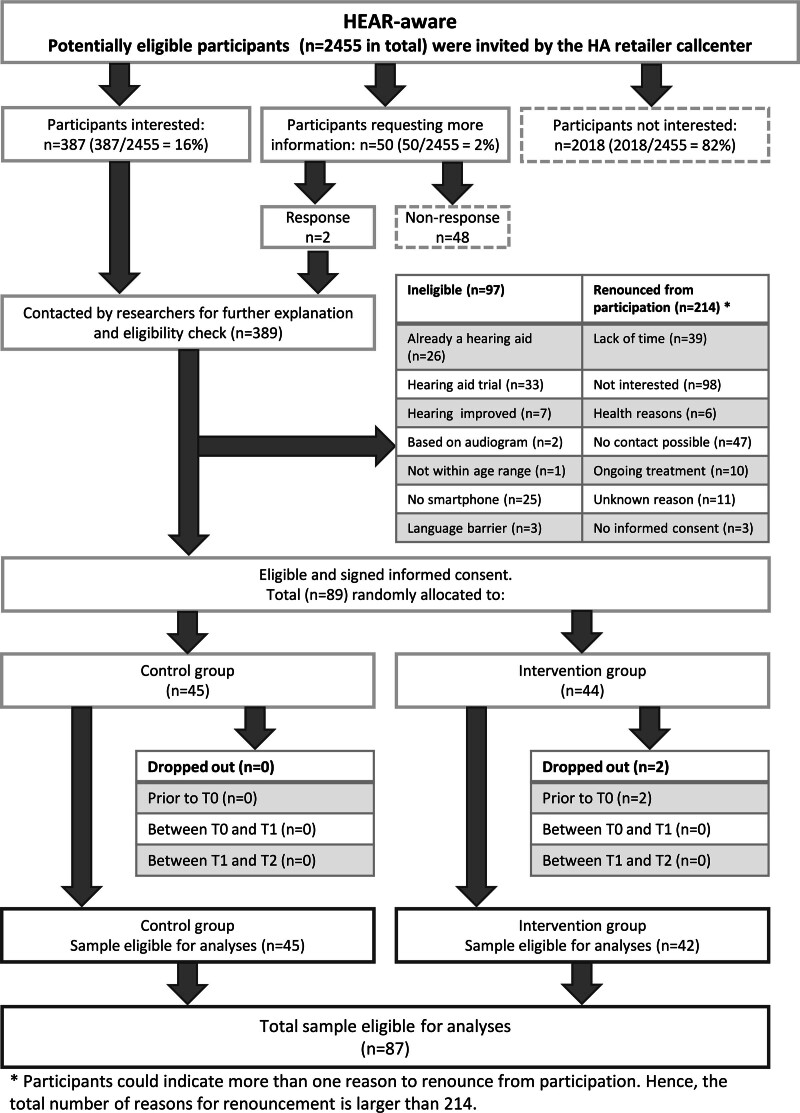
Participants’ flow through the study.

Depending on the listening situation that was added (see Figure 1, screenshot 2 for examples in Supplemental Digital Content, http://links.lww.com/EANDH/B424), certain acoustic labels were applied, triggering the release of educational snippets (the EMI element of the app). Examples of labels are “Speech in noise,” “Speech in quiet,” “Speech listening media, for example, TV/radio,” and “Telephone conversation.” The snippets included videos, texts, and web links on several themes, that in turn corresponded to certain TBs (see Figure 1, screenshot 3 in Supplemental Digital Content, http://links.lww.com/EANDH/B424). These themes (and TBs) were: (1) background knowledge on hearing (no TB); (2) communication strategies (TB communication strategies); (3) coping with impaired hearing (TB emotional coping); (4) understanding by loved ones (TB social support); (5) hearing at work (no TB); (6) ALDs (TB ALDs), (7) HAs (TB HAs); (8) Fun (no TB). The theme “fun” consisted of published columns and articles about sound(s) or hearing.

In total, 118 snippets were available, of which 81 were released in response to the participant reporting on a new listening situation. Each snippet was released only once for each participant. After their release, snippets became part of the app’s library. Thirty-seven snippets were already available in the library from the start and could always be accessed. The participants had the opportunity to share each snippet with their family or friends via a share icon. Some descriptive statistics (e.g., number and type of added listening situations) and survey-question-answers were provided to the user to facilitate hearing difficulty awareness raising. This was possible under the icon “My Statistics” where users could also compare their statistics to those of other app users.

Users were also asked to give a quick review of the snippets they had opened. Because all data were updated real-time, users could also see mean review scores based on all users’ input. Further details of the HEAR-aware app content have been described in Pronk et al. (2024). App usage was monitored via the online CMS. Pronk et al. defined the following criteria for “high” compliance: (1) add at least 1 to 2 (i.e., ≥1.5) listening situations per day across 4 weeks which equals to 42 in total; (2) evaluate ≥80% of the added situations; (3a) view ≥80% of offered snippets, (3b) resulting in 33 snippets in total, and (4) view ≥10 standard library snippets.

### Recruitment and Inclusion Criteria

Recruitment and data collection were conducted between May and October 2021. Participants in this study were adults with hearing loss aged 50 years and over who had visited an HA retailer (Schoonenberg HoorSupport) in the past 1.5 years for a hearing test-appointment or a subsequent intake-appointment. They were candidates for HAs (see inclusion criterion 2), but had chosen not to pursue this option. They were approached via a call center employee of Schoonenberg HoorSupport and were briefly informed about this study. Those who expressed interest received a patient information letter. After confirming their willingness to participate, a researcher called the participant to provide any further information about the study and check the eligibility criteria. Inclusion criteria for the study were:

1. Age 50 years or older.
2. Minimum pure tone threshold of 35 dB HL averaged over 1, 2, and 4 kHz in at least one ear. This is the minimum threshold for Dutch health care insurance companies to reimburse the HA costs at a minimum of 75%, or in full depending on the participant’s insurance provider. This way, financial constraints are minimized as the main reason for not taking up HAs and HA “eligibility” is ensured. Note that over-the-counter HAs are not available in the Netherlands.
3. Visited an HA retailer for a hearing test-appointment or a subsequent intake-appointment in the past 1.5 years, but decided to not pursue an HA trajectory. Note that a hearing test-appointment and subsequent intake-appointments are free of cost in the Netherlands and hence, participants had not paid for an initial evaluation.
4. Still does not (yet) want an HA.
5. Never tried an HA before.
6. Owns an e-mail account and a smartphone or tablet and uses apps.
7. Fluent in Dutch.
8. Willing to use the app on a daily basis.

Participants were excluded when their main complaint was tinnitus or when participants were enrolled in care provided by a specialized audiology clinic.

### Parameters/Outcome Measures

In our previous study (Pronk et al. 2024), we examined the psychometric properties of variants of two different types of target behavior (TB)-specific readiness outcomes: The Line (yielding a continuous outcome) and the Staging Algorithm (yielding a categorical outcome). Although both measures showed satisfactory test-retest reliability and construct validity, The Line was considered more sensitive because of its continuous response scale. Hence, it was adopted as the primary outcome measure for Objective 1 of the present study. The Staging Algorithm was included as one of the secondary outcome measures (see later).

### Primary Outcome Measure

The Line is a discrete 11-point scale ranging from 0 (not ready at all) to 10 (highly ready) (Rollnick et al. 1999; Tønnesen 2012; Ingo et al. 2017). The original The Line is generic in nature and asks “How important is it to improve your hearing right now?” ranging from 0 (not important at all) to 10 (highly important). We adapted the tool to address the concept of readiness more explicitly and created five sub-versions that measured readiness specifically for the five separate TBs addressed in the app. These items read as follows: (1) “How ready are you to apply communication strategies in your daily life?,” (2) “How ready are you to work on your feelings about your diminished hearing?” (emotional coping), (3) “How ready are you to involve others in your diminished hearing?” (social support), (4) “How ready are you to try hearing aids?” and (5) “How ready are you to try assistive listening devices?.” Items were accompanied by a short explanation and examples. For each of them, participants indicated their readiness on a discrete 11-point scale ranging from 0 (not ready at all) to 10 (highly ready) at T0, T1, and T2. For each measurement moment, we calculated the average of the five TB-specific The Line ratings, which we further refer to as The Line Composite score. This was adopted as the primary outcome of this study. The five TB-specific sub-versions of The Line were also examined. These were treated as secondary outcomes.

### Secondary Outcome Measures

Secondary outcome measures, additional to the five TB-specific sub-versions of The Line, are listed later. Moments of administration for all questionnaires are shown in Table 1 in Supplemental Digital Content, http://links.lww.com/EANDH/B425.

1. The Line Generic: this outcome measure assessed generic readiness to take action on hearing by asking: “How ready are you to work on your diminished hearing?” This measure is based on The Line as used by Ingo et al. (2017). It has a discrete 11-point scale ranging from 0 (not ready at all) to 10 (highly ready).
2. Dutch, adapted version of the Staging Algorithm (Milstein & Weinstein 2002): this outcome measure assesses generic readiness to take action on hearing loss. It consists of one question: “Which of the following statements best describes your view on your current hearing status?” Participants indicated which of the four possible answers best represented their thinking about generic “taking action on their hearing.” The response categories represented different degrees (i.e., stages) of readiness: 1 (precontemplation), 2 (contemplation), 3 (preparation) or 4 (action). For the five different TBs (applying communication strategies, improving emotional coping, facilitating social support, taking up HAs, and taking up ALDs) both the question and response categories were adjusted to suit the TB. As an example, the item on improving emotional coping was: “Which of the following statements best describes your current view on working on your feelings about your diminished hearing?” with the following response categories: (1) “I don’t think my hearing problem is such that working on my feelings would be of any help”; (2) “I think I have a hearing problem where working on my feelings may be helpful, I am just not ready yet to put them into practice now, but I might be in the future”; (3) “I know I have a hearing problem where working on my feelings could be helpful, and I intend to do this soon”; (4) “I know I have a hearing problem where working on my feelings is helpful, and therefore I am already working on this.”
3. The Partners in Health Scale: this scale (modified for hearing loss) was translated into Dutch for the purpose of this study. It assesses hearing loss self-management (Convery et al. 2018) and covers six items addressing self-management on knowledge (two items), recognition and management of symptoms (two items), and coping (two items). Items could be rated from 0 (low self-management) to 8 (high self-management). Scores were averaged for all questions yielding a total score. For each of the three subscales, scores were averaged as well.
4. The 28-item Amsterdam Inventory for Auditory Disability and Handicap (Kramer et al. 1995): it covers five subscales addressing Distinction of Sounds (eight items), Auditory Localization (five items), Intelligibility in Noise (five items), Intelligibility in Quiet (five items) and Detection of Sounds (five items). It has a four-point Likert scale with response categories being: 0 (almost always), 1 (frequently), 2 (occasionally), 3 (almost never). The overall mean score of all 28 items was included in the analysis as well as subscale means. These could range from 0 to 3, with higher scores indicating greater disability.
5. Dutch version of the Communication Profile for the Hearing Impaired (CPHI): this instrument assesses coping behavior (Mokkink et al. 2010). It has six subscales: Maladaptive Behaviors (seven items), Verbal Strategies (seven items), Non-Verbal Strategies (five items), Self-Acceptance (four items), Acceptance of Loss (three items), and Stress and Withdrawal (nine items). The five-point response scale (ranging from 1 to 5) was either a frequency continuum or an agree–disagree continuum, depending on the question. For all subscales, scores were averaged and could range from 1 to 5 with higher scores indicating better coping.
6. The 29-item Attitude Questionnaire (AQ): this instrument addresses attitudes on hearing loss and HAs (Van den Brink et al. 1996; Pronk et al. 2017). It covers five subscales, namely Benefits of HAs (10 items), Hearing Loss Stigma (6 items), Sound Quality and Cost of HAs (3 items), Social Pressure and Support (5 items), Evaluation of HAs by Others (3 items) and Geriapathy (2 items). Items were scored from 1 (strongly disagree) to 5 (strongly agree) and averaged per subscale. Higher scores indicated a more positive attitude toward the particular topic.
7. The Self-Efficacy for Hearing Help-Seeking Scale: this scale covers four statements asking to what degree respondents felt confident to: “choose a health care professional for their hearing problems,” “arrange an appointment,” “physically get to this appointment,” “succeed in making an appointment happen.” The response scale for each statement ranged from 0 (low) to 100 (high self-efficacy). Scores of the four items were averaged.
8. Prior Hearing Help-Seeking Steps Scale (PHHSS): this scale asked whether or not participants had taken help-seeking steps before the particular measuring moment (T0, T1, T2). The steps were: “performed an online hearing self-test” (before T0, T1, T2), “visited a health care provider for their hearing” (before T0, T1, T2) or “had a trial period and possibly purchased an HA or an ALD” (before T1, T2).

To address objective 2 of this study, the following outcome measures were used. Please note that these were administered at T1 to the intervention group only.

9. The SUS (Brooke 1996): it provides an evaluation of usability of a system or device. It comprises 10 items, scored from 0 to 4. The summed total is multiplied by 2.5. Therefore, the total score can range from 0 (worst imaginable) to 100 (best imaginable) (Bangor et al. 2008). According to Bangor et al. (2008) SUS scores less than 50 should be considered a cause for significant concern and are judged to be unacceptable. Products with scores between 50 and 70 should be considered candidates for increased scrutiny and continued improvement and should be judged to be marginal at best. Products which are at least passable have SUS scores >70 with better products scoring in the high 70s to upper 80s. Truly superior products score better than 90.
10. The Value/Usefulness subscale of the Intrinsic Motivation Inventory (IMI, Deci & Ryan 1985): it assessed how useful the participant found the app. The scale comprises seven statements each with a seven-point Likert rating scale with anchor points 1 (not at all true), 4 (somewhat true), and 7 (very true).
11. Item 4 of the International Outcome Inventory-Alternative Intervention (IOI-AI; (Kramer et al. 2002); Noble 2002): this was used as an indicator of an individual’s overall satisfaction with the app. The item reads: “Considering everything, do you think the app is worth the trouble?” It has five response options with higher scores indicating better outcomes: 1 (not at all), 2 (slightly), 3 (moderately), 4 (quite a lot) and 5 (very much worth it).
12. Product recommendation item: this was used to assess overall satisfaction. The item asked: “How likely is it that you would recommend the app to other people (family, friends, colleagues)?” Response options could range from 0 (not at all likely) to 10 (extremely likely), see also Meijerink et al. (2017).

### Internal Consistency of the Questionnaires

Internal consistency of all questionnaires was assessed. Cronbach α coefficient was calculated for instruments with at least three items, while the Spearman–Brown coefficient was computed for two-item questionnaires. Three scales exhibited an internal consistency of ≤0.6 and were consequently excluded from the analyses: the AQ Sound Quality and Cost of HAs (α coefficient: 0.4), AQ Geriapathy (Spearman–Brown coefficient: 0.2) and the CPHI Acceptance of Loss (α coefficient: 0.6).

### Sample Size

To gain a relevant TB-specific readiness difference of 1.5 points between groups postintervention on the primary outcome (The Line Composite), it was calculated that each arm should include 37 participants which means 74 in total (independent-samples *t* test, pooled SD = 2.55, two-sided *α* = 0.05, *β* = 0.80). The chosen SD and effect size were informed by the study of Pronk et al. (2024). Anticipating a 10% drop-out rate, a total of 84 participants (42 per group) would be needed. The number of n = 37 (intervention group) was considered sufficient to address the second objective of this study too.

### Randomization and Blinding

Participants were randomized in a 1:1 ratio on an individual level without stratification, using variable block randomization (of sizes 4 and 6) in Castor EDC. Randomization was done before the T0 baseline measurement. Hence, at T0 participants were aware of the group to which they were allocated. Blinding of participants was not possible, because the intervention group was provided access to the app after completing the first round of questionnaires whereas the control group did not receive an intervention. The researchers could not be blinded as they needed to distribute partially distinct sets of questionnaires to the two groups and assist intervention group participants in downloading the app.

### Statistical Analysis

To assess the comparability of baseline participant characteristics between both groups, independent-samples *t* tests were conducted for normally distributed continuous variables, Mann–Whitney U tests for nonnormally distributed continuous variables, and Chi-square tests for dichotomous and categorical variables.

For objective 1, we applied Linear Mixed effect Models with group (control/intervention), time (T0/T1/T2) and the Group × Time interaction as fixed effects and subjects as random effects. This was done for the primary and secondary outcomes, except for the Prior Hearing Help Seeking scale for which a Chi-square test was used to compare groups. Normality was checked for all outcomes by visually inspecting histograms of all variable distributions. For none of them a transformation was necessary or yielded an improved normally distributed variable. Analyses were performed on intention-to-treat (ITT) basis, including all participants. Any participant characteristics that would appear to be significantly different between groups at baseline would be considered potential confounders and added as covariates in the models, but this did not apply (see Results). Partly completed questionnaires were also included in the analyses, causing some variation in the number of participants included in the different Linear Mixed effect Model analyses. Imputation of missing outcomes was not considered necessary as Linear Mixed Modeling is known to be robust for missing data.

In case of significant group differences over time of an outcome, post-hoc comparisons were performed to investigate between which measurement moments (T1 or T2) the change from T0 differed between groups. Post-hoc comparisons were only conducted for the ITT analyses. This was done using the estimated fixed effects with Bonferroni correction. We decided not to lower the *p* values (Bonferroni correction) for multiple comparisons in the main ITT readiness analyses, nor for the secondary outcomes, but only for the post-hoc analyses. The reason is that secondary measures normally do not require stricter *p* values (Rothman 1990). These are usually only applied to (multiple) primary outcome measures. In addition, target-behavior-specific “readiness” is a new construct in our area of research. To the best of our knowledge, this is the first exploration of various (adapted) TB-specific versions of The Line and the Staging Algorithm as intervention outcome measures. Therefore, these analyses can be regarded as “exploratory” in nature and too stringent multiple testing correction in exploratory research may result in potentially effective interventions be abandoned (Wason et al. 2014).

Compliance with app use was determined based on the number of listening situations added and the number of snippets viewed per participant. CMS data indicated that app usage varied considerably among participants in the intervention group. The total number of listening situations added varied from 0 to 114 per participant across the 4 weeks. All predefined listening situations were reported at least once. Table 2 in Supplemental Digital Content, http://links.lww.com/EANDH/B426, presents the frequency of types of listening situations reported. The number of snippets viewed per participants ranged from 0 to 75 with a mean of 17 snippets viewed per participant. To investigate whether more favorable effects were observed in participants who used the app more frequently, we conducted an additional per protocol (PP) analysis. In this analysis, we compared the PP intervention group to the control group. Inclusion criteria for the intervention PP group were based on Pronk et al. (2024) who found that 65% of their participants met at least one of the following criteria:

- At least 13 entered listening situations in combination with at least 10 viewed snippets or
- At least four entered listening situations in combination with at least 30 viewed snippets.

These cutoffs were a compromise between a “high” intervention dose received (see paragraph “Intervention” for a definition) and a cutoff that would result in a not too low number of participants to be able to run the statistical analyses.

To address objective 2, we calculated descriptive statistics for the SUS, IMI, IOI-AI item 4, and the recommendation item. Statistical analyses were performed in SPSS version 28. The two-sided significance level was set at 0.05.

## RESULTS

### Participants

Figure 1 shows the participants’ flow through the study. In total, 389 potential participants initially expressed interest in the study, but 300 of them either withdrew from participation or were found to be ineligible. The remaining 89 participants provided informed consent and were randomly assigned to either the intervention group (n = 44) or the control group (n = 45). Two participants from the intervention group did not complete the baseline survey and were subsequently excluded from further analyses, leading to a total of 87 participants (42 in the intervention group and 45 in the control group). Participant characteristics are shown in Table 1.

**TABLE 1.** Baseline characteristics of all participants included in the Linear Mixed Models analyses (n = 87)

Variable	Control (n = 45)	Intervention (n = 42)	*p*
Hearing loss*, mean (SD)	44.0 (12.1)	42.9 (7.8)	0.63†
Tinnitus (yes)	16 (35.6%)	19 (45.2%)	0.36‡
Age in years, mean (SD), range	68.3 (8.7), 51–90	64.8 (9.1), 48§–84	0.067†
Female	10 (22.2%)	16 (38.1%)	0.11‡
Marital status			0.81‡
Married	34 (75.6%)	32 (76.2%)	
Cohabiting	5 (11.1%)	2 (4.8%)	
Widow or widower	1 (2.2%)	1 (2.4%)	
Divorced	3 (6.7%)	4 (9.5%)	
Single, never married	2 (4.4%)	3 (7.1%)	
Living situation			0.90‡
Living together with other people	39 (86.7%)	36 (85.7%)	
Living alone	6 (13.3%)	6 (14.3%)	
Level of education			0.073‡
Low	1 (2.2%)	3 (7.1%)	
Middle	40 (88.9%)	29 (69.0%)	
High	4 (8.9%)	10 (23.8%)	
Occupational status (paid job, yes)	14 (31.1%)	21 (50.0%)	0.073‡
Comorbidity (yes)	25 (55.6%)	18 (42.9%)	0.24‡

Means (SD) and counts (%) are presented.

* Poorer ear average in dB HL, averaged across 1, 2, and 4 kHz.

† Independent-samples *t* test.

‡ Chi-square test.

§ After enrollment in the study, two participants appeared to be slightly younger than 50, namely 48 and 49 yrs old respectively. We decided to keep them in the study.

published online ahead of print June 04, 2024

No significant differences were observed between the intervention and control groups (*p* > 0.05) in terms of participant characteristics (Table 1). In addition, the primary and secondary outcome measures were evenly distributed within each group at baseline (*p* > 0.05), see Table 3 in Supplemental Digital Content, http://links.lww.com/EANDH/B427.

### Objective 1, ITT Analyses

**Fig. 2.**
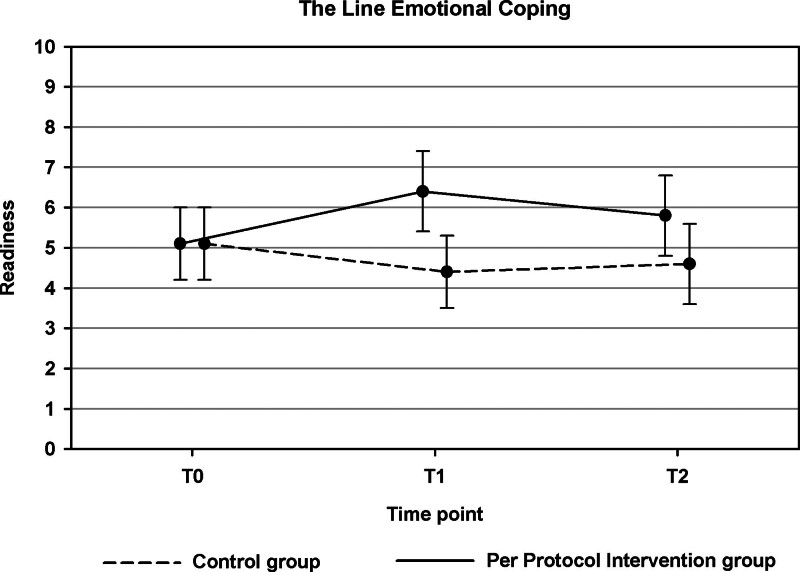
Results of the intention-to-treat analyses for The Line Emotional Coping (objective 1). Estimated scores at T0, T1, and T2 are shown for the intervention group and control group. Error bars indicate 95% confidence intervals.

Table 2 shows the results of the ITT analyses. There was no significant difference between the groups for the primary outcome measure, The Line Composite (*p* = 0.12). Nonetheless, a Significant Group × Time Interaction effect was identified for one of the specific outcomes related to The Line TB, namely, The Line Emotional Coping (*p* = 0.033). This effect indicated a higher readiness to cope with emotional feelings about hearing problems in the intervention group than in the control group (Fig. 2). The post-hoc analysis revealed that the estimated difference in change between the two groups was significant between T0 and T1 (*p* = 0.009) (an estimated improvement of 1.26 point in the intervention group and an estimated deterioration of 0.52 in the control group), but not between T2 and T0 (*p* = 0.19). In the intent-to-treat (ITT) analyses, none of the remaining outcomes exhibited significant group differences (*p* > 0.05, see Tables 2 and 3).

**TABLE 2.** Effectiveness results for the primary and secondary outcomes (intention-to-treat analyses, objective 1)

		T0		T1		T2		
Outcome	Group	n	Mean (SD)	n	Mean (SD)	n	Mean (SD)	LMM*
The Line Composite	Control	43	5.2 (2.1)	38	4.6 (2.0)	39	5.0 (2.4)	0.12
	Intervention	42	5.2 (2.1)	28	5.6 (1.9)	34	5.7 (1.8)	
The Line Communication Strategies	Control	43	5.7 (2.6)	38	4.9 (2.9)	39	5.2 (3.0)	0.056
	Intervention	42	5.6 (2.6)	28	6.4 (2.4)	34	6.2 (2.4)	
The Line Emotional Coping	Control	43	5.1 (2.8)	38	4.4 (2.9)	39	4.6 (3.3)	**0.033**
	Intervention	42	5.1 (2.9)	28	6.4 (2.6)	34	5.8 (2.9)	
The Line Social Support	Control	43	5.2 (2.8)	38	4.7 (2.9)	39	4.9 (3.2)	0.77
	Intervention	42	5.5 (2.7)	28	5.6 (2.9)	34	5.8 (2.8)	
The Line Hearing Aids	Control	43	5.3 (2.7)	38	5.4 (3.1)	39	6.3 (3.0)	0.19
	Intervention	42	5.5 (2.5)	28	5.6 (3.1)	34	5.7 (2.6)	
The Line Assistive Listening Devices	Control	43	4.5 (2.7)	38	3.6 (3.1)	39	4.0 (3.2)	0.14
	Intervention	42	4.2 (2.7)	28	4.3 (2.6)	34	4.8 (2.4)	
The Line Generic	Control	43	6.2 (2.2)	38	6.0 (2.1)	40	6.6 (2.2)	0.30
	Intervention	42	6.7 (2.0)	28	6.5 (1.9)	34	6.6 (1.7)	
Staging Algorithm Generic	Control	43	1.5 (0.7)	38	1.5 (0.7)	40	1.7 (0.9)	0.66
	Intervention	42	1.6 (0.7)	28	1.7 (0.8)	34	1.7 (0.8)	
Staging Algorithm Communication Strategies	Control	43	1.2 (1.1)	38	1.1 (1.2)	39	1.0 (1.1)	0.52
	Intervention	42	1.3 (1.2)	28	1.5 (1.4)	34	1.6 (1.2)	
Staging Algorithm Emotional Coping	Control	43	0.6 (1.0)	38	0.5 (0.9)	39	0.9 (1.2)	0.40
	Intervention	42	1.0 (1.2)	28	1.1 (1.3)	34	1.2 (1.4)	
Staging Algorithm Social Support	Control	43	0.8 (1.0)	38	0.8 (1.1)	39	1.2 (1.3)	0.96
	Intervention	42	1.2 (1.3)	28	1.5 (1.3)	34	1.6 (1.3)	
Staging Algorithm Hearing Aids	Control	43	1.1 (0.6)	38	1.3 (0.8)	39	1.5 (0.9)	0.18
	Intervention	42	1.3 (0.7)	28	1.3 (0.7)	34	1.4 (0.9)	
Staging Algorithm Assistive Listening Devices	Control	43	0.9 (0.8)	38	0.7 (0.8)	39	0.6 (0.8)	0.13
	Intervention	42	0.7 (0.8)	28	0.8 (0.7)	34	0.9 (0.8)	
PHS self-management total	Control	43	6.0 (1.3)	38	6.1 (1.2)	39	6.3 (1.3)	0.28
	Intervention	42	5.9 (1.3)	28	6.5 (0.9)	34	6.6 (1.0)	
PHS knowledge	Control	43	5.4 (1.6)	38	5.5 (1.7)	39	5.9 (1.4)	0.35
	Intervention	42	5.4 (1.6)	28	6.1 (1.1)	34	6.4 (1.1)	
PHS management of symptoms	Control	43	5.8 (2.0)	38	6.0 (1.9)	39	6.3 (1.8)	0.77
	Intervention	42	5.5 (2.0)	28	6.1 (1.8)	34	6.3 (1.7)	
PHS coping	Control	43	6.7 (1.4)	38	6.9 (1.3)	39	6.6 (1.7)	0.22
	Intervention	42	6.7 (1.4)	28	7.1 (0.8)	34	7.1 (1.0)	
CPHI maladaptive behaviors	Control	44	4.6 (0.4)	40	4.6 (0.4)	40	4.6 (0.4)	0.57
	Intervention	42	4.7 (0.3)	28	4.6 (0.3)	34	4.6 (0.4)	
CPHI verbal strategies	Control	44	2.3 (0.8)	40	2.3 (0.9)	40	2.3 (0.8)	0.18
	Intervention	42	2.2 (0.6)	28	2.3 (0.8)	34	2.5 (0.8)	
CPHI nonverbal strategies	Control	44	2.9 (1.0)	40	3.0 (1.0)	40	3.0 (0.9)	0.16
	Intervention	42	3.0 (0.9)	28	2.9 (0.8)	34	3.2 (0.9)	
CPHI self-acceptance	Control	44	4.4 (0.7)	40	4.4 (0.6)	40	4.5 (0.5)	0.83
	Intervention	42	4.5 (0.6)	28	4.5 (0.5)	34	4.5 (0.6)	
CPHI stress and withdrawal	Control	44	4.0 (0.7)	39	4.1 (0.6)	40	4.0 (0.8)	0.84
	Intervention	42	4.1 (0.6)	28	4.2 (0.6)	34	4.1 (0.6)	
SEHHS	Control	43	82.1 (17.2)	38	83.2 (13.4)	39	85.7 (10.0)	0.12
	Intervention	42	87.5 (13.5)	28	86.8 (12.4)	34	85.3 (15.8)	
AQ benefits of hearing aids	Control	43	3.6 (0.5)	38	3.6 (0.4)	40	3.6 (0.5)	0.41
	Intervention	42	3.7 (0.6)	28	3.6 (0.5)	34	3.6 (0.6)	
AQ hearing loss stigma	Control	43	2.2 (0.9)	38	2.2 (0.8)	40	2.2 (0.7)	0.82
	Intervention	42	2.5 (0.9)	28	2.5 (0.9)	34	2.4 (0.9)	
AQ social pressure and support	Control	43	3.0 (0.9)	38	3.0 (0.9)	40	3.1 (0.9)	0.15
	Intervention	42	3.1 (0.7)	28	3.0 (0.8)	34	3.0 (0.9)	
AQ evaluation of hearing aids by others	Control	43	2.4 (0.7)	38	2.3 (0.5)	40	2.3 (0.6)	0.45
	Intervention	42	2.3 (0.7)	28	2.1 (0.7)	34	2.2 (0.8)	
AIADH total	Control	44	0.7 (0.5)	40	0.6 (0.4)	41	0.7 (0.5)	0.17
	Intervention	42	0.7 (0.4)	28	0.7 (0.4)	34	0.7 (0.4)	
AIADH distinction of sounds	Control	44	0.5 (0.4)	40	0.4 (0.4)	41	0.5 (0.5)	0.63
	Intervention	42	0.4 (0.4)	28	0.5 (0.4)	34	0.5 (0.5)	
AIADH auditory localization	Control	44	0.7 (0.6)	40	0.6 (0.6)	41	0.7 (0.6)	0.58
	Intervention	42	0.7 (0.7)	28	0.7 (0.8)	34	0.7 (0.6)	
AIADH intelligibility in noise	Control	44	1.2 (0.6)	40	1.1 (0.6)	41	1.2 (0.7)	0.080
	Intervention	42	1.3 (0.5)	28	1.2 (0.5)	34	1.2 (0.5)	
AIADH intelligibility in quiet	Control	44	0.8 (0.6)	40	0.8 (0.6)	41	0.8 (0.5)	0.23
	Intervention	42	0.9 (0.4)	28	0.9 (0.5)	34	0.9 (0.5)	
AIADH detection of sounds	Control	44	0.6 (0.5)	40	0.5 (0.4)	41	0.6 (0.5)	0.057
	Intervention	42	0.5 (0.4)	28	0.6 (0.4)	34	0.5 (0.4)	

Significant effects are indicated in bold.

* *p* value of the interaction between time and group.

AIADH, Amsterdam Inventory for Auditory Disability and Handicap; AQ, Attitude Questionnaire; CPHI, Communication Profile for the Hearing Impaired; LMM, Linear Mixed Model; PHS, Partners in Health Scale; SEHHS, Self-Efficacy for Hearing Help-Seeking Scale.

**TABLE 3.** Effectiveness results for the secondary outcome: prior hearing help-seeking steps (intention-to-treat analyses, part of objective 1)

		T1 and/or T2	Count (%)	
Variables	Group	n		*p*
Visited a healthcare provider, yes	Control	40	15 (37.5)	0.18*
	Intervention	34	18 (52.9)	
Started a trial period for an HA/ALD, yes	Control	41	3 (7.3)	1.00†
	Intervention	40	3 (7.5)	
Performed a hearing selftest, yes	Control	41	6 (14.6)	0.31*
	Intervention	38	9 (23.7)	

* Chi-square test.

† Fisher exact test.

ALD, alternative listening devices; HA, hearing aids.

### Objective 1, PP Analyses

In total, 21 of the 42 participants in the intervention group met the criteria for inclusion in the PP analyses. There were no significant differences between the PP group and the not-PP group (*p* > 0.05) for any of the participant characteristics listed in Table 1.

**Fig. 3.**
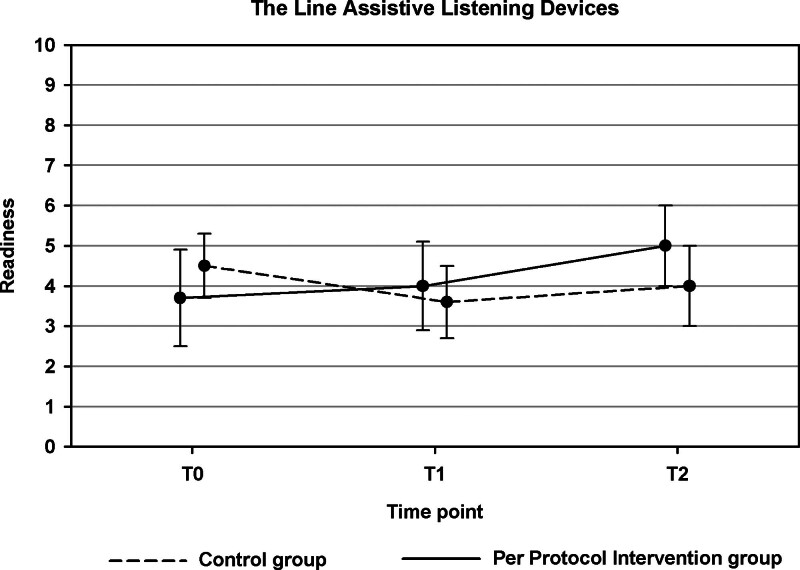
Results of the per protocol analyses for The Line Assistive Listening Devices (part of objective 1). Estimated scores are shown for the per protocol intervention group and control group. Error bars indicate 95% confidence intervals.

**Fig. 4.**
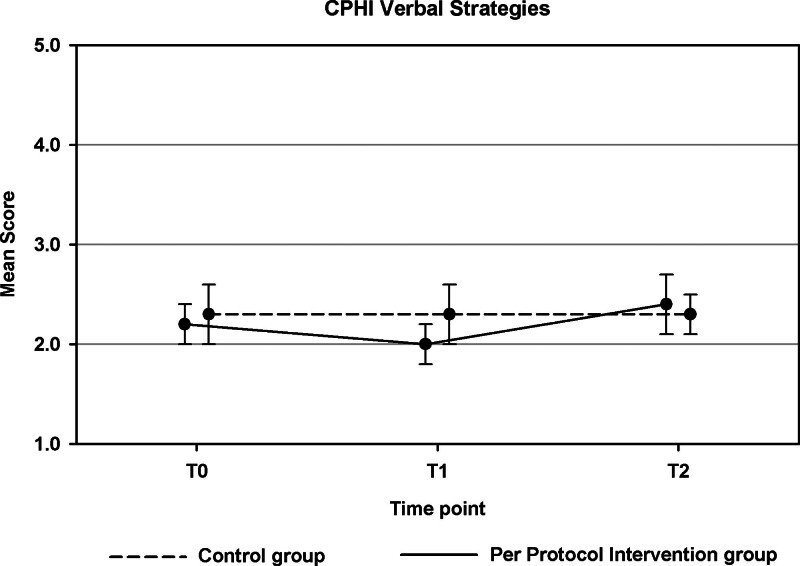
Results of the per protocol analyses for the Communication Profile for the Hearing Impaired Verbal Strategies (part of objective 1). Estimated scores are shown for the per protocol intervention group and control group. Error bars indicate 95% confidence intervals.

For three secondary outcomes, the course of outcomes across T0, T1, and T2 differed significantly between the PP intervention and the control group (see Table 4 in Supplemental Digital Content, http://links.lww.com/EANDH/B428). First, this was the case for the Line ALDs (*p* = 0.049, Fig. 3). Whereas an overall decrease in readiness to take up ALDs was observed in the control group, an increase in readiness was found for the PP intervention group. Second, there was a significant group difference in the pattern of change across T0, T1, and T2 for the CPHI Verbal Strategies (*p* = 0.030). This effect is illustrated in Figure 4.

**Fig. 5.**
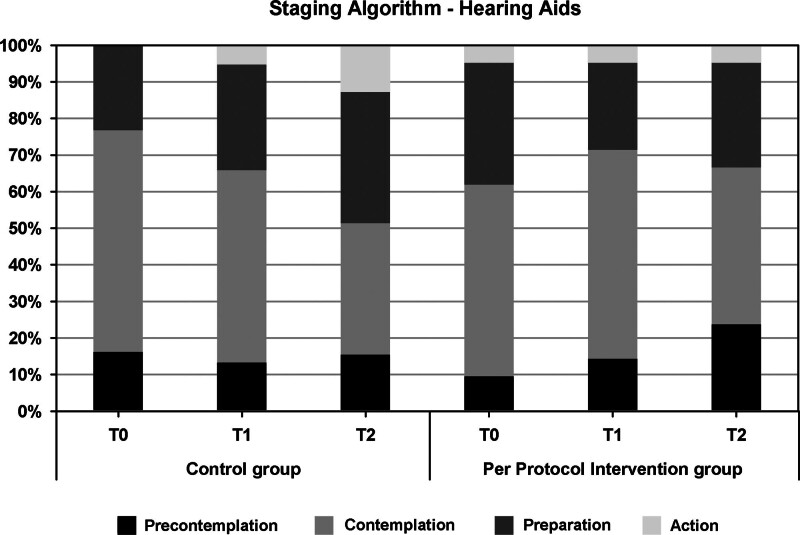
Results of the Per Protocol analyses for the Staging Algorithm HAs (part of objective 1). Stages of Change scores (proportions) are shown for Per Protocol intervention group and control group at the three time points (T0, T1, and T2). HAs indicate hearing aids.

Lastly, the pattern of change in the Staging Algorithm HAs score differed over time for the two groups (*p* = 0.049, Fig. 5). There was a trend toward: (1) an increasing number of participants in the precontemplation stage over time in the PP intervention group mainly at the expense of the number of contemplators. And (2) an increasing number of participants in the action and preparation stages over time in the control group (at the expense of the number of participants in the contemplation stage). None of the remaining outcomes in the PP analyses showed significant group differences (*p* > 0.05).

### Objective 2

Directly after app use (T1), overall usability (SUS), usefulness (IMI), and satisfaction (IOI-item 4) were measured in the intervention group. Scores are shown in Table 4. SUS scores ranged from 40 to 90 with an average of 67.5 points (SD = 14.9), indicating “marginal” usability. The categorical variable showed that about half of the participants scored the app’s usability as good (>70 points) while a minority (n = 5) scored it as unacceptable (≤50 points, see Table 4). The average IMI score was 4.5 (SD = 0.9). The median IOI-Item 4 score was 3 (moderately worth it) and the median score for the recommendation item was 6 on a scale ranging from 0 to 10.

**TABLE 4.** Overall usability (SUS) and overall usefulness (IMI) of the HEAR-aware app according to the Intervention group at T1 (objective 2)

Outcome	n	Score
SUS total score, mean (SD) 0 worst imaginable—100 best imaginable	30	67.5 (14.9)
SUS total—categorical (%)	30	
Unacceptable (scores ranged from 40.0 to 47.5)		5 (16.7%)
Marginable (scores ranged from 55.0 to 70.0)		11 (30.0%)
Good (scores ranged from 71 to 90)		14 (33.3%)
IMI, Mean (SD) 1 lowest usefulness—7 highest usefulness	28	4.5 (1.0)
IOI-AI item 4, Median (25th and 75th percentiles)	28	3 (2.0–3.0)
Recommendation item, Median (25th and 75th percentiles)	28	6 (3.3–8.0)

IMI, Intrinsic Motivation Inventory; IOI-AI, International Outcome Inventory-Alternative Intervention; SUS, System Usability Scale.

## DISCUSSION

### Objective 1: Effectiveness

In this study, we investigated the effectiveness of the HEAR-aware smartphone app for adults who were eligible for HAs, had chosen not to pursue them (yet), but were open to receiving support on various TBs. The app was specifically designed to assist adults in self-managing issues or challenges related to their hearing loss. This was operationalized through the use of the primary outcome measure The Line Composite score, reflecting general “readiness to take action” which was averaged across five TBs, namely “applying communication strategies,” “improving emotional coping,” “seeking social support,” “uptake of hearing aids,” and “uptake of ALDs.” Effects on readiness for each of the individual TBs were examined too.

A priori, we hypothesized that individuals in the intervention group would demonstrate an increased readiness to take action on these TBs. This hypothesis was not supported for the primary The Line Composite outcome, neither in the ITT analysis, nor in the PP analysis. However, the ITT analyses demonstrated a significant difference for one of the TB-specific measures of The Line, that is, for Emotional Coping. Participants in the intervention group reported a greater readiness to address their emotional responses related to hearing problems, such as learning to accept them and reducing negative emotions, following the intervention phase, as compared with the control group. Their readiness for emotional coping increased from 5.1 to 6.4 points (on a scale from 0 to 10) between T0 and T1. Validated cutoff scores for The Line do not exist. However, Ratanjee-Vanmali et al. (2019) used a cutoff score of greater than 5 on the generic “The Line” scale as a criterion for offering advice regarding the subsequent steps in the hearing help-seeking process. Furthermore, participants were required to have a Staging Algorithm score of 3 or 4 to qualify for additional evaluation. This approach was intended to ensure an adequate level of readiness for seeking help and initiating an HA trial. It should be noted that Ratanjee-Vanmali et al. used a generic version of “The Line” and examined the TB “uptake of HAs” only, so it is difficult to directly compare the results and draw inferences for our emotional coping results. Within our control group, the readiness for emotional coping decreased from 5.1 to below the midpoint of the scale at 4.6 points. Conversely, the mean score at T1 in our intervention group was 6.4, significantly exceeding the midpoint of the scale, which is an encouraging result.

Emotional coping was one of the themes in the app for which snippets were created. These included videos, texts, and web links. Two examples of snippets where emotional coping was the primary TB are: “Sharing your hearing problems with others, a good idea?” and “Dealing with hearing challenges – You and your conversation partner.” These included testimonials of individuals with hearing impairment who shared their experiences related to their hearing difficulties and help-seeking journey. Five of the 118 snippets in total had “Readiness to work on feelings about diminishing hearing ability” (emotional coping) as the main TB. This number was comparable to the number of snippets on the other themes, except HAs. It is therefore unlikely that the number of emotional coping snippets alone can explain the significant finding on readiness for emotional coping in our study. Other app elements may have contributed too such as snippets touching upon components related to emotional coping (e.g., snippets addressing “Experiences with hearing aids”), thus indirectly facilitating readiness.

In addition, it is possible that the act of completing the EMA surveys heightened participants’ awareness of their hearing problems, subsequently fostering their readiness to address the emotional aspects related to the acceptance of hearing loss. While the present study did not yield any significant effects on hearing disability (Amsterdam Inventory for Auditory Disability and Handicap), which does not align with this hypothesis, it is noteworthy that a majority of the participants in Pronk et al. (2024) expressed that completing the EMA surveys had offered them a valuable “greater insight into their hearing.”

Another important point to note is that both the CPHI (subscales Self-Acceptance, Acceptance of Loss, Stress, and Withdrawal) and the Partners in Health Scale emotional coping subscale failed to demonstrate significant differences between the intervention and control group. These measures were designed to assess the practical application of various emotional coping behaviors in participants’ daily lives. This implies that the app may have primarily enhanced participants’ readiness or intention to take action in the domain of emotional coping, rather than directly improving the actual application of emotional coping strategies. According to several social psychological models and theories, someone’s intentions precede the initiation of behavioral change (Sheeran 2002). A stronger intention to engage in a particular behavior makes it more likely that the behavior will be carried out. Therefore, the app may be a facilitator of crucial initial steps in the hearing help-seeking journey, helping individuals establish the intention to take action. It is worthwhile to explore this further in future research. For example, one may want to investigate to what extent the increased readiness to take action, stemming from the use of apps like HEAR-aware, translates into sustainable behavioral changes (e.g., uptake of HAs, better coping, uptake of ALDs, see later) over the long term.

As shown in Figure 2, readiness for emotional coping in the intervention group decreased slightly from 6.4 to 5.9 during the 4-week period after the end of app use. One may wonder whether follow-up rehabilitation directly after the end of a period of app use would be useful. Further research is needed to identify whether and what kind of follow-up interventions would be suitable. Relevant to mention in this context is a recent study by Bennett et al. (2021) who invited consumer and community representatives to think about how the provision of support for clients experiencing emotional and psychological issues in relation to their hearing loss might be improved. The three behaviors voted by the participants to be the most promising for a behavioral intervention included the clinician (a) asking about, (b) providing information on, and (c) delivering therapeutic intervention for emotional and psychological well-being within audiological service provision. In their study, Bennett et al. addressed benefits of in-person follow-up rather than e- or m-health or virtual follow-up. Nonetheless, use of apps like HEAR-aware may be considered helpful to facilitate support for the emotional and psychological issues that arise relating to hearing loss. Further research is needed to investigate to what extent apps like HEAR-aware can replace or support human interaction in the aural rehabilitation process.

Surprisingly, there was no significant difference between the PP intervention group and the control group for TB-specific readiness for emotional coping. The PP intervention group size was small (n = 21). This number may have been too small to detect a significant difference. However, the PP analyses did reveal a significant difference between the PP intervention and control group for The Line ALDs (Fig. 3). Changes in readiness to take up ALDs across the T0 to T3 interval showed an overall increase in readiness in the PP intervention group while a decrease was observed in the control group. Interestingly, an increase in readiness to take up ALDs across T0 to T3 in the intervention group was also observed with The Line ALD (ITT) (Table 2). This was not significant, but at least confirms consistency in outcome. We speculate that this finding might relate to the results obtained with the Staging Algorithm HAs (Fig. 5) which showed that participants in the PP intervention group were slightly less ready to take up HAs than the control group at T2. It is possible that the information on ALDs provided in the app raised users’ awareness of the existence of ALDs. This is consistent with Southall et al. (2006) who demonstrated that “awareness that technical solutions other than HAs exist” is one of the landmarks to see people moving toward successful ALD use. Heightened awareness in the present study might have contributed to participants’ increased readiness to consider ALDs as a viable alternative to HAs. While we assume that many factors influencing HA uptake such as “cost” and “stigma” may also influence ALD uptake, another explanation may relate to the fact that most ALDs are designed for specific activities (e.g., watching television, conversations over the phone), while HAs are intended for general-purpose use (Southall et al. 2006). Participants may have felt a need for better hearing in specific activities only. There was however no difference in the number of participants in either group who had actually started an HA or ALD trial period (Table 3). The follow-up period of 4 weeks may have been insufficient to observe tangible effects on the actual uptake of devices. Longer term follow-up results need to be investigated.

A significant difference between the PP intervention group and the control group was also found for the CPHI Verbal Strategies scale (Fig. 4). The pattern of change from T0, T1 to T2 was slightly different for the two groups. Whereas the control group remained stable over time, the PP intervention group score decreased slightly and then increased again. Overall scores in both groups remained fairly low (<3) though. It is possible that the 4-week follow-up period was too short to detect a further increase in the utilization of verbal strategies.

### Objective 2: Overall Usefulness, Usability, and Satisfaction

Participants rated the app as moderately useful (mean IMI score of 4.5 on a scale from 1 to 7), moderately worth it (IOI-item 4) and its overall usability (SUS) as “marginal” (mean = 67.5 points). Distribution of the SUS data (Table 4) indicated that about half of the participants rated the app as good. A minority (5 participants) rated it as “unacceptable.” Similarly, scores on the recommendation item varied widely ranging from 3.3 to 8.0 (scale 0 to 10) with a mean of 6. Similar results, including a wide range in scores and a dichotomy was also observed in our previous study (Pronk et al. 2024). Apparently, there were participants who liked the tool and who did not like it at all. This is relevant information for the implementation of self-management tools like the HEAR-aware app in (digital) care pathways. We were unable to investigate the underlying reasons for this contrast in usability and satisfaction ratings, as we did not conduct a process evaluation study, which is addressed in the Limitations. It is debatable whether individuals who were employed had less time to utilize the app. This factor may have played a role in the wide range of usability scores observed and in the absence of more significant differences between the control and the intervention group. There were however no differences in participant characteristics, including occupational status, in the PP group (47.6% employed) and the not-PP group (52.4% employed) which makes it less likely that occupational status had an important role in the outcomes observed. The same holds for other participant characteristics like marital status and living situation. Nevertheless, the observed range of usability scores indicate that tailoring implementation to the end-user’s needs and desires seems essential.

### Strengths

A notable strength of this study was its RCT design, which included two postintervention measurement points. This approach allowed for the assessment of effects not only immediately after the intervention period but also at a later stage. Another strength concerns the type of intervention: a unique smartphone application designed for adults with hearing loss who are not ready for an HA. Also, some novel outcome measures were developed specifically for this study. Rather than only focusing on readiness to take up HAs, “readiness to take action” on a range of different TBs was assessed, all of which are recognized as relevant for hearing loss self-management across the hearing help-seeking journey (i.e., applying communication strategies, improving emotional coping, seeking social support, and taking up ALDs). A recent study demonstrated that hearing care professionals are generally positive toward embedding EMA-type smartphone apps in audiological care (Galvin et al. 2022). The professionals reported that such apps could offer valuable information to support their counseling of clients, enhance clients’ understanding of their hearing challenges, and empower them to self-manage their hearing conditions. These findings underline the supportive role EMA/EMI that apps can play in hearing rehabilitation. Lastly, for the construct of readiness, we used various outcome measures, including adapted versions of both The Line and the Staging Algorithm. We conducted a thorough assessment of the internal consistency of all our questionnaires and included only those with satisfactory internal consistency in our analysis.

### Limitations

There are a number of limitations to this study that need to be mentioned. First, there was no alternative intervention for the control group, such as a sham app, to make it a double-blinded RCT study. Blinding of participants and researchers was not possible and this may have biased the results. Due to resource constraints, we were unable to develop or use an alternative intervention such as a sham app in the control group. In the intervention group, utilization of the HEAR-aware app and the attention and guidance provided by the researchers on how to use the app among participants in the intervention group, could have influenced participants’ scores on the questionnaires, independently of the app’s content. This warrants further investigation in future research. Furthermore, at enrollment participants were informed that if they would be allocated to the control group, they would receive the HEAR-aware app after completion of the study. This was done to promote study enrollment and prevent-drop out. However, this knowledge can have biased the controls’ responses during the study, potentially influencing the study results.

In addition, it is often advised to conduct a process evaluation study in conjunction with an RCT (Moore et al. 2015) to determine whether the effectiveness or lack thereof of an intervention is attributable to implementation-related factors or the intervention itself. Due to resource constraints, we were unable to conduct a process evaluation study. Such a study, including conducting interviews with app users, could have been valuable in elucidating the range in outcomes related to the app’s usability and user satisfaction, and how these insights could be practically applied, such as in the targeted provision of the app. Follow-up research is essential to discern why the HEAR-aware app proved successful for certain individuals while failing for others. Such research can elucidate the distinctions between (un)successful implementation and intervention outcomes and highlight aspects of the HEAR-aware app that may need to be improved to enhance overall effectiveness. Note that at the start of the study, all participants had shown interest in the app intervention and consented to participate.

Furthermore, the follow-up time in this study was rather short. Any long-term effects (i.e., >4 weeks) remain unknown and deserve to be investigated in future research.

Last, we performed a large number of statistical tests. We are aware that some significant effects may have been found by chance and hence, outcomes should be interpreted with caution. Nonetheless, the results of this study may provide valuable insights for shaping the design and the choice of readiness outcome measures in future studies like this one.

## CONCLUSIONS

The utilization of the HEAR-aware app resulted in an increase in participants’ readiness to address the emotional aspects related to their hearing problems, specifically in terms of emotional coping. Given that a lack of readiness to take action on hearing issues is commonly recognized as a significant barrier in the hearing help-seeking journey, including intervention uptake, this outcome is indeed promising. Notably, among participants who used the app more frequently, there was a heightened readiness to consider ALDs, whereas the readiness to consider HAs did not show improvement. It is hypothesized that the app’s information on ALDs may have raised awareness among users about their existence, thereby enhancing their willingness to consider ALDs as an alternative to HAs. Furthermore, participants exhibited both positive and negative reactions to the app, suggesting that it may not be a suitable intervention for everyone. This study underscores the potential of self-management support tools like the HEAR-aware app in the rehabilitation of adults with hearing loss who are not yet ready for HAs.

## ACKNOWLEDGMENTS

The authors thank Dr. Ariane Laplante-Lévesque for her collaboration and advice in the design of the Self-Efficacy for Hearing Help-Seeking Scale (SEHHS). The authors are very grateful for the indispensable help from Schoonenberg HoorSupport, via Vera Jansen, Dorinde van Santen, Rencia Stortenbeker, and Lars de Ruiter in the recruitment of participants for this study. EverywhereIM built the app and we are grateful for their active and professional role in this study. The authors thank VeiligheidNL for allowing the use of the book “101 vragen over horen,” and Schoonenberg HoorSupport for allowing the use of the “HoorSupport” program, for snippet content.

## Supplementary Material


