# Supplementary material for: Effectiveness of the HEAR-Aware App for Adults Not Ready for Hearing Aids, but Open to Self-Management Support: Results of a Randomized Controlled Trial
Source: Ear Hear. 2024 Jun 4;45(6):1502–16. doi: 10.1097/AUD.0000000000001533 (PMC11487041; doi:10.1097/AUD.0000000000001533)

Supplemental Digital Content (SDC)

SDC Figure 1: **A selection of screenshots of the HEAR-aware app**

Screenshot 1

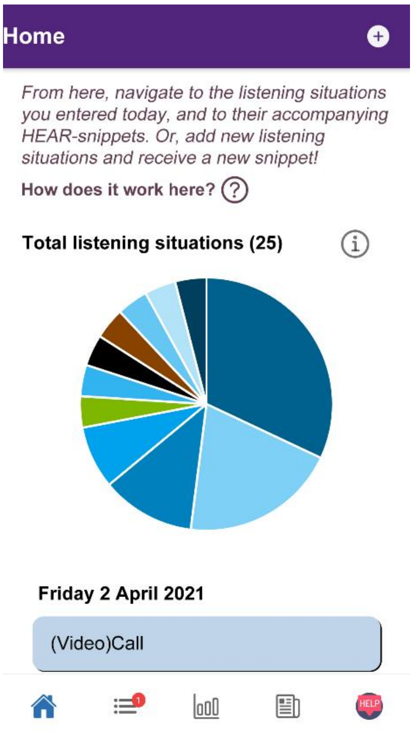

Screenshot 2

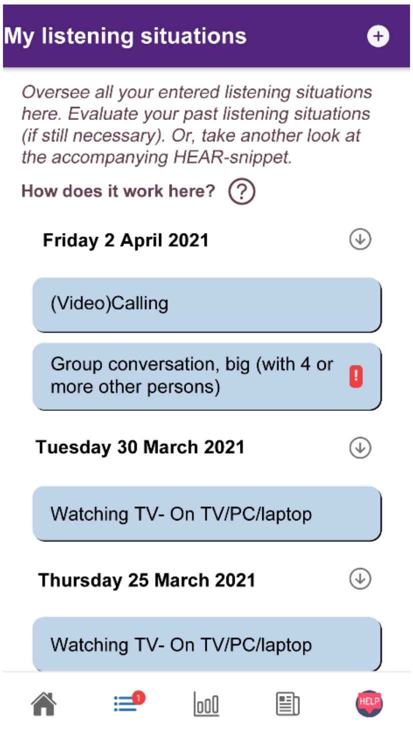

Screenshot 3

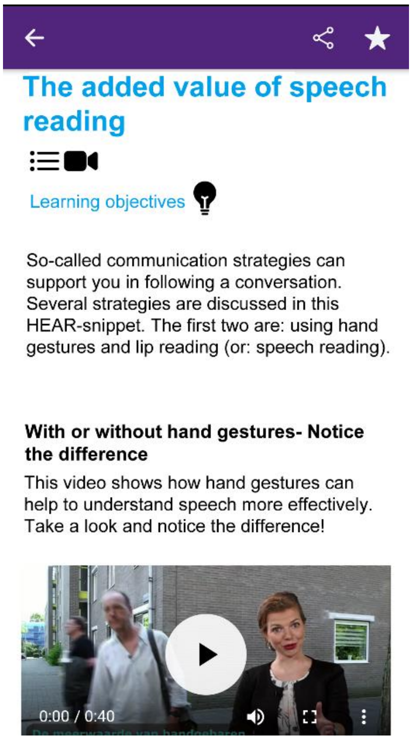

Supplement: Supplementary file 1 [file aud-45-1502-s001.pdf]
