# Supplementary material for: Effectiveness of the HEAR-Aware App for Adults Not Ready for Hearing Aids, but Open to Self-Management Support: Results of a Randomized Controlled Trial
Source: Ear Hear. 2024 Jun 4;45(6):1502–16. doi: 10.1097/AUD.0000000000001533 (PMC11487041; doi:10.1097/AUD.0000000000001533)
Supplement: Supplementary file 2 [file aud-45-1502-s002.pdf]

Supplemental Digital Content (SDC)

SDC Table 1: **Overview of all questionnaires and time points at which they were administered.**

	Pre-intervention questionnaire (T0) Intervention & Control Group	Post-intervention questionnaire 1 (T1) Control Group	Post-intervention questionnaire 1 (T1) Intervention Group	Post-intervention questionnaire 2 (T2) Control Group	Post-intervention questionnaire 2 (T2) Control Group
Demographics	X				
The Line	X	X	X	X	X
Staging Algorithm	X	X	X	X	X
AIADH	X	X	X	X	X
CPHI	X	X	X	X	X
PHS	X	X	X	X	X
AQ	X	X	X	X	X
SEHHS	X	X	X	X	X
PHHSS	X	X	X	X	X
SUS			X		
IMI			X		
IOI-AI item 4			X		X
Recommendation item			X		

AIADH = Amsterdam Inventory for Auditory Disability and Handicap, AQ = Attitude Questionnaire, CPHI = Communication Profile for the Hearing Impaired, IMI = Intrinsic Motivation Inventory (Value/Usefulness subscale), IOI-AI = International Outcome Inventory-Alternative Intervention, PHHSS = Prior Hearing Help-Seeking Steps, PHS = Partners in Health Scale, Recommendation item = 'How likely is it that you would recommend the app to other people (family, friends, colleagues)?', SEHHS = Self-Efficacy for Hearing Help-Seeking Scale, SUS = System Usability Scale,
