# Supplementary material for: Effectiveness of the HEAR-Aware App for Adults Not Ready for Hearing Aids, but Open to Self-Management Support: Results of a Randomized Controlled Trial
Source: Ear Hear. 2024 Jun 4;45(6):1502–16. doi: 10.1097/AUD.0000000000001533 (PMC11487041; doi:10.1097/AUD.0000000000001533)
Supplement: Supplementary file 3 [file aud-45-1502-s003.pdf]

Supplemental Digital Content (SDC)

SDC Table 2. The frequency of different types of listening situations reported by the participants in the intervention group (those who utilized the HEAR-aware app).

**Type of Listening Situation**	**Number of Listening Situations Added (Frequency)**	**Percentage of Listening Situations Entered (Top-5 Indicated in Bold)**
1. **1-on-1 conversation**	149	**18.3**
2. **Group conversation, small (with 2-3 others)**	126	**15.5**
3. **Watching TV – On TV/PC/laptop**	119	**14.6**
4. **Group conversation, big (with ≥ 4 others)**	84	**10.3**
5. **(Video)calling**	62	**7.6**
6. Driving car	44	5.4
7. Music	33	4.0
8. Walking	31	3.8
9. Cycling	30	3.7
10. Listening to nature sounds (e.g., birds, sea)	29	3.6
11. Lecture/ presentation	29	3.6
12. Sports instruction	16	2.0
13. Watching clips – On smartphone	12	1.5
14. Radio interview / podcast / audiobook	14	1.7
15. Using public transport	7	.9
Other	30	3.7
**Total**	**815**	**100.0**
