# Supplementary material for: Effectiveness of the HEAR-Aware App for Adults Not Ready for Hearing Aids, but Open to Self-Management Support: Results of a Randomized Controlled Trial
Source: Ear Hear. 2024 Jun 4;45(6):1502–16. doi: 10.1097/AUD.0000000000001533 (PMC11487041; doi:10.1097/AUD.0000000000001533)
Supplement: Supplementary file 4 [file aud-45-1502-s004.pdf]

Supplemental Digital Content (SDC)

SDC Table 3. **Baseline comparison of outcome measures. Values indicate the mean (and SD) of the outcomes unless stated otherwise. Significant effects are indicated in bold.**

Baseline characteristics			
Outcome measures	Control (n=45)	Intervention(n=42)	p-value
Primary outcome measure			
The Line Composite	5.2 (2.1)	5.2 (2.1)	.95[a]
Secondary outcome measures			
The Line			
The Line Communication Strategies	5.7 (2.6)	5.6 (2.6)	.89[a]
The Line Emotional Coping	5.1 (2.8)	5.1 (2.9)	.94[a]
The Line Social Support	5.2 (2.8)	5.5(2.7)	.57[a]
The Line Hearing Aids	5.3 (2.6)	5.5 (2.5)	.82[a]
The Line Assistive Listening Devices	4.5 (2.7)	4.2 (2.7)	.62[a]
The Line Generic	6.2 (2.2)	6.7 (2.0)	.25[a]
Staging Algorithm. Count (%)			
Staging Algorithm Generic			.55[c]
Precontemplation	2 (4.7%)	0 (0%)	
Contemplation	23 (53.5%)	23 (54.8%)	
Preparation	14 (32.6%)	14 (33.3%)	
Action	4 (9.3%)	5 (11.9%)	
Staging Algorithm Communication Strategies			.37[c]
Precontemplation	16 (37.2%)	13 (31.0%)	
Contemplation	10 (23.3%)	13 (31.0%)	
Preparation	11 (25.6%)	6 (14.3%)	
Action	6 (14.0%)	10 (23.8%)	
Staging Algorithm Emotional Coping			.30[c]
Precontemplation	29 (67.4%)	22 (52.4%)	
Contemplation	4 (9.3%)	6 (14.3%)	
Preparation	7 (16.3%)	6 (14.3%)	
Action	3 (7.0%)	8 (19.0%)	
Staging Algorithm Social Support			.13[c]
Precontemplation	23 (53.5%)	18 (42.9%)	
Contemplation	10 (23.3%)	9 (21.4%)	
Preparation	6 (14.0%)	3 (7.1%)	
Action	4 (9.3%)	12 (28.6%)	
Staging Algorithm Hearing Aids			.44[c]
Precontemplation	7 (16.3%)	5 (11.9%)	
Contemplation	26 (60.5%)	23 (54.8%)	
Preparation	10 (23.3%)	12 (28.6%)	
Action	0 (0.0%)	2 (4.8%)	
Staging Algorithm Assistive Listening Devices			.40[c]
Precontemplation	15 (34.9%)	19 (45.2%)	
Contemplation	17 (39.5%)	16 (38.1%)	
Preparation	11 (25.6%)	6 (14.3%)	
Action	0 (0.0%)	1 (2.4%)	
PHS			
PHS Total	6.0 (1.3)	5.9 (1.3)	.78[a]
PHS Knowledge	5.4 (1.6)	5.4 (1.6)	.84[a]
PHS Symptoms	5.8 (2.0)	5.5 (2.0)	.56[a]
PHS Coping	6.7 (1.4)	6.7 (1.4)	.89[a]
AIADH			
AIADH Total	0.7 (0.5)	0.7 (0.4)	.79[a]
AIADH Distinction of Sounds	0.5 (0.4)	0.4 (0.4)	.54[a]
AIADH Auditory Localization	0.7 (0.6)	0.7 (0.7)	.81[a]

AIADH Intelligibility in Noise	1.2 (0.6)	1.3 (0.5)	.24[a]
AIADH Intelligibility in Quiet	0.8 (0.6)	0.9 (0.4)	.39[a]
AIADH Detection of Sounds	0.6 (0.5)	0.5 (0.4)	.65[a]
CPHI			
CPHI Maladaptive Behaviors	4.6 (0.4)	4.7 (0.3)	.29[a]
CPHI Verbal Strategies	2.3 (0.8)	2.2 (0.6)	.77[a]
CPHI Non-Verbal Strategies	2.9 (1.0)	3.0 (0.9)	.88[a]
CPHI Self-Acceptance	4.4 (0.7)	4.5 (0.6)	.54[a]
CPHI Stress and Withdrawal	4.0 (0.7)	4.1 (0.6)	.38[a]
AQ			
AQ Benefits of Hearing Aids	3.6 (0.5)	3.7 (0.6)	.68[a]
AQ Hearing Loss Stigma	2.2 (0.9)	2.5 (0.9)	.18[a]
AQ Social Pressure and Support	3.0 (0.9)	3.1 (0.7)	.65[a]
AQ Evaluation of Hearing Aids by Others	2.4 (0.7)	2.3 (0.7)	.44[a]
SEHHS. Median (25th – 75th percentile)			
Self efficacy for hearing help seeking total	85.0 (70.0 – 95.3)	92.5 (79.3 –98.6)	.11[b]
PHHSS. Count (%)			
Selftest Yes	10 (22.2%)	11 (26.2%)	.67[c]

[a] Independent Samples Test, [b] Mann-Whitney test, [c] Chisquare test.

Abbreviations: AIADH = Amsterdam Inventory for Auditory Disability and Handicap, AQ = Attitude Questionnaire, CPHI = Communication Profile for the Hearing Impaired, PHHSS = Prior Hearing Help-Seeking Steps, PHS = Partners in Health Scale, SEHHS = Self-Efficacy for Hearing Help-Seeking Scale,

SDC Table 4. **Effectiveness results for the primary and secondary outcomes (per-protocol analyses). Significant effects are indicated in bold.**

**Outcomes**		**T0**		**T1**		**T2**		**LMM[a]**
	**Group**	**n**	**Mean (SD)**	**n**	**Mean (SD)**	**n**	**Mean (SD)**	
The Line Composite	Control	43	5.2 (2.1)	38	4.6 (2.0)	39	5.0 (2.4)	.23
	Per Protocol	21	5.1 (2.0)	21	5.4 (1.9)	21	5.4 (1.7)	
The Line Communication Strategies	Control	43	5.7 (2.6)	38	4.9 (2.9)	39	5.2 (3.0)	.13
	Per Protocol	21	5.7 (2.6)	21	6.3 (2.6)	21	5.9 (2.6)	
The Line Emotional Coping	Control	43	5.1 (2.8)	38	4.4 (2.9)	39	4.6 (3.3)	.11
	Per Protocol	21	5.2 (2.7)	21	6.2 (2.6)	21	5.2 (2.9)	
The Line Social Support	Control	43	5.2 (2.8)	38	4.7 (2.9)	39	4.9 (3.2)	.90
	Per Protocol	21	5.5 (2.5)	21	5.3 (3.0)	21	5.6 (2.8)	
The Line Hearing Aids	Control	43	5.3 (2.7)	38	5.4 (3.1)	39	6.3 (3.0)	.26
	Per Protocol	21	5.3 (2.6)	21	5.4 (3.3)	21	5.3 (2.5)	
The Line Assistive Listening Devices	Control	43	4.5 (2.7)	38	3.6 (3.1)	39	4.0 (3.2)	**.049**
	Per Protocol	21	3.7 (2.8)	21	4.0 (2.6)	21	5.0 (2.3)	
The Line Generic	Control	43	6.2 (2.2)	38	6.0 (2.1)	40	6.6 (2.2)	.13
	Per Protocol	21	6.8 (2.0)	21	6.3 (1.9)	21	6.2 (1.7)	
Staging Algorithm Generic	Control	43	1.5 (0.7)	38	1.5 (0.7)	40	1.7 (0.9)	.37
	Per Protocol	21	1.6 (0.8)	21	1.7 (0.8)	21	1.6 (0.7)	
Staging Algorithm Communication Strategies	Control	43	1.2 (1.1)	38	1.1 (1.2)	39	1.0 (1.1)	.80
	Per Protocol	21	1.9 (1.0)	21	1.7 (1.3)	21	1.7 (1.2)	
Staging Algorithm Emotional Coping	Control	43	0.6 (1.0)	38	0.5 (0.9)	39	0.9 (1.2)	.26
	Per Protocol	21	1.0 (1.2)	21	1.2 (1.3)	21	1.1 (1.3)	
Staging Algorithm Social Support	Control	43	0.8 (1.0)	38	0.8 (1.1)	39	1.2 (1.3)	.55
	Per Protocol	21	1.5 (1.3)	21	1.5 (1.3)	21	1.5 (1.3)	
Staging Algorithm Hearing Aids	Control	43	1.1 (0.6)	38	1,3 (0.8)	39	1.5 (0.9)	**.049**
	Per Protocol	21	1.3 (0.7)	21	1.2 (0.8)	21	1.1 (0.9)	

Staging Algorithm Assistive Listening Devices	Control	43	0.9 (0.8)	38	0.7 (0.8)	39	0.6 (0.8)	.38
	Per Protocol	21	0.7 (0.7)	21	0.8 (0.6)	21	0.7 (0.7)	
PHS Self Management total	Control	43	6.0 (1.3)	38	6.1 (1.2)	39	6.3 (1.3)	.47
	Per Protocol	21	6.1 (1.1)	21	6.6 (0.9)	21	6.8 (0.8)	
PHS Knowledge	Control	43	5.4 (1.6)	38	5.5 (1.7)	39	5.9 (1.4)	.44
	Per Protocol	21	5.5 (1.5)	21	6.1 (1.1)	21	6.5 (1.1)	
PHS Management of Symptoms	Control	43	5.8 (2.0)	38	6.0 (1.9)	39	6.3 (1.8)	.79
	Per Protocol	21	6.0 (1.8)	21	6.5 (1.6)	21	6.9 (1.1)	
PHS Coping	Control	43	6.7 (1.4)	38	6.9 (1.3)	39	6.6 (1.7)	.74
	Per Protocol	21	7.0 (1.2)	21	7.2 (0.8)	21	7.1 (1.1)	
CPHI Maladaptive behaviors	Control	44	4.6 (0.4)	40	4.6 (0.4)	40	4.6 (0.4)	.78
	Per Protocol	21	4.7 (0.3)	21	4.6 (0.3)	21	4.6 (0.3)	
CPHI Verbal Strategies	Control	44	2.3 (0.8)	40	2.3 (0.9)	40	2.3 (0.8)	**.03**
	Per Protocol	21	2.2 (0.5)	21	2.0 (0.4)	21	2.4 (0.7)	
CPHI Non-Verbal Strategies	Control	44	2.9 (1.0)	40	3.0 (1.0)	40	3.0 (0.9)	.45
	Per Protocol	21	3.1 (0.7)	21	2.9 (0.7)	21	3.1 (0.7)	
CPHI Self-Acceptance	Control	44	4.4 (0.7)	40	4.4 (0.6)	40	4.5 (0.5)	.54
	Per Protocol	21	4.5 (0.7)	21	4.6 (0.5)	21	4.5 (0.7)	
CPHI Stress and Withdrawal	Control	44	4.0 (0.7)	39	4.1 (0.6)	40	4.0 (0.8)	.72
	Per Protocol	21	4.1 (0.5)	21	4.2 (0.6)	21	4.1 (0.6)	
SEHHS Self efficacy for hearing help seeking	Control	43	82.1 (17.2)	38	83.2 (13.4)	39	85.7 (10.0)	.52
	Per Protocol	21	88.2 (15.3)	21	86.2 (13.5)	21	88.5 (13.4)	
AQ Benefits	Control	43	3.6 (0.5)	38	3.6 (0.4)	40	3.6 (0.5)	.07
	Per Protocol	21	3.7 (0.6)	21	3.5 (0.5)	21	3.4 (0.5)	
AQ Stigma	Control	43	2.2 (0.9)	38	2.2 (0.8)	40	2.2 (0.7)	.66
	Per Protocol	21	2.5 (0.8)	21	2.4 (0.8)	21	2.3 (0.8)	
AQ Social Pressure	Control	43	3.0 (0.9)	38	3.0 (0.9)	40	3.1 (0.9)	.08
	Per Protocol	21	3.0 (0.7)	21	2.8 (0.7)	21	2.8 (0.8)	
AQ Evaluation of Hearing Aids by Others	Control	43	2.4 (0.7)	38	2.3 (0.5)	40	2.3 (0.6)	.28
	Per Protocol	21	2.3 (0.8)	21	2.1 (0.7)	21	2.3 (0.9)	
AIADH Total	Control	44	0.7 (0.5)	40	0.6 (0.4)	41	0.7 (0.5)	.35
	Per Protocol	21	0.7 (0.3)	21	0.7 (0.3)	21	0.7 (0.4)	
AIADH Distinction of Sounds	Control	44	0.5 (0.4)	40	0.4 (0.4)	41	0.5 (0.5)	.53
	Per Protocol	21	0.3 (0.3)	21	0.4 (0.3)	21	0.5 (0.5)	
AIADH Auditory Localization	Control	44	0.7 (0.6)	40	0.6 (0.6)	41	0.7 (0.6)	.80
	Per Protocol	21	0.6 (0.5)	21	0.6 (0.5)	21	0.6 (0.5)	
AIADH Intelligibility in Noise	Control	44	1.2 (0.6)	40	1.1 (0.6)	41	1.2 (0.7)	.05
	Per Protocol	21	1.2 (0.5)	21	1.2 (0.5)	21	1.1 (0.5)	
AIADH Intelligibility in Quiet	Control	44	0.8 (0.6)	40	0.8 (0.6)	41	0.8 (0.5)	.18
	Per Protocol	21	0.9 (0.4)	21	0.8 (0.3)	21	0.8 (0.5)	
AIADH Detection of Sounds	Control	44	0.6 (0.5)	40	0.5 (0.4)	41	0.6 (0.5)	.11
	Per Protocol	21	0.5 (0.3)	21	0.6 (0.3)	21	0.5 (0.3)	

[a] p-value of the interaction between time and group.
SD = Standard deviation, LMM = Linear Mixed Model. AIADH = Amsterdam Inventory for Auditory Disability and Handicap, AQ = Attitude Questionnaire, CPHI = Communication Profile for the Hearing Impaired, PHHSS = Prior Hearing Help-Seeking Steps, PHS = Partners in Health Scale, SEHHS = Self-Efficacy for Hearing Help-Seeking Scale,
