# Supplementary material for: Effectiveness of the HEAR-Aware App for Adults Not Ready for Hearing Aids, but Open to Self-Management Support: Results of a Randomized Controlled Trial
Source: Ear Hear. 2024 Jun 4;45(6):1502–16. doi: 10.1097/AUD.0000000000001533 (PMC11487041; doi:10.1097/AUD.0000000000001533)
Supplement: Supplementary file 5 [file aud-45-1502-s005.pdf]

SDC Table 4. **Effectiveness results for the primary and secondary outcomes (per-protocol analyses). Significant effects are indicated in bold.**

Outcomes		T0		T1		T2		LMM[a]
	Group	n	Mean (SD)	n	Mean (SD)	n	Mean (SD)	
The Line Composite	Control	43	5.2 (2.1)	38	4.6 (2.0)	39	5.0 (2.4)	.23
	Per Protocol	21	5.1 (2.0)	21	5.4 (1.9)	21	5.4 (1.7)	
The Line Communication Strategies	Control	43	5.7 (2.6)	38	4.9 (2.9)	39	5.2 (3.0)	.13
	Per Protocol	21	5.7 (2.6)	21	6.3 (2.6)	21	5.9 (2.6)	
The Line Emotional Coping	Control	43	5.1 (2.8)	38	4.4 (2.9)	39	4.6 (3.3)	.11
	Per Protocol	21	5.2 (2.7)	21	6.2 (2.6)	21	5.2 (2.9)	
The Line Social Support	Control	43	5.2 (2.8)	38	4.7 (2.9)	39	4.9 (3.2)	.90
	Per Protocol	21	5.5 (2.5)	21	5.3 (3.0)	21	5.6 (2.8)	
The Line Hearing Aids	Control	43	5.3 (2.7)	38	5.4 (3.1)	39	6.3 (3.0)	.26
	Per Protocol	21	5.3 (2.6)	21	5.4 (3.3)	21	5.3 (2.5)	
The Line Assistive Listening Devices	Control	43	4.5 (2.7)	38	3.6 (3.1)	39	4.0 (3.2)	**.049**
	Per Protocol	21	3.7 (2.8)	21	4.0 (2.6)	21	5.0 (2.3)	
The Line Generic	Control	43	6.2 (2.2)	38	6.0 (2.1)	40	6.6 (2.2)	.13
	Per Protocol	21	6.8 (2.0)	21	6.3 (1.9)	21	6.2 (1.7)	
Staging Algorithm Generic	Control	43	1.5 (0.7)	38	1.5 (0.7)	40	1.7 (0.9)	.37
	Per Protocol	21	1.6 (0.8)	21	1.7 (0.8)	21	1.6 (0.7)	
Staging Algorithm Communication Strategies	Control	43	1.2 (1.1)	38	1.1 (1.2)	39	1.0 (1.1)	.80
	Per Protocol	21	1.9 (1.0)	21	1.7 (1.3)	21	1.7 (1.2)	
Staging Algorithm Emotional Coping	Control	43	0.6 (1.0)	38	0.5 (0.9)	39	0.9 (1.2)	.26
	Per Protocol	21	1.0 (1.2)	21	1.2 (1.3)	21	1.1 (1.3)	
Staging Algorithm Social Support	Control	43	0.8 (1.0)	38	0.8 (1.1)	39	1.2 (1.3)	.55
	Per Protocol	21	1.5 (1.3)	21	1.5 (1.3)	21	1.5 (1.3)	
Staging Algorithm Hearing Aids	Control	43	1.1 (0.6)	38	1,3 (0.8)	39	1.5 (0.9)	**.049**
	Per Protocol	21	1.3 (0.7)	21	1.2 (0.8)	21	1.1 (0.9)	
Staging Algorithm Assistive Listening Devices	Control	43	0.9 (0.8)	38	0.7 (0.8)	39	0.6 (0.8)	.38
	Per Protocol	21	0.7 (0.7)	21	0.8 (0.6)	21	0.7 (0.7)	
PHS Self Management total	Control	43	6.0 (1.3)	38	6.1 (1.2)	39	6.3 (1.3)	.47
	Per Protocol	21	6.1 (1.1)	21	6.6 (0.9)	21	6.8 (0.8)	
PHS Knowledge	Control	43	5.4 (1.6)	38	5.5 (1.7)	39	5.9 (1.4)	.44
	Per Protocol	21	5.5 (1.5)	21	6.1 (1.1)	21	6.5 (1.1)	
PHS Management of Symptoms	Control	43	5.8 (2.0)	38	6.0 (1.9)	39	6.3 (1.8)	.79
	Per Protocol	21	6.0 (1.8)	21	6.5 (1.6)	21	6.9 (1.1)	
PHS Coping	Control	43	6.7 (1.4)	38	6.9 (1.3)	39	6.6 (1.7)	.74
	Per Protocol	21	7.0 (1.2)	21	7.2 (0.8)	21	7.1 (1.1)	
CPHI Maladaptive behaviors	Control	44	4.6 (0.4)	40	4.6 (0.4)	40	4.6 (0.4)	.78
	Per Protocol	21	4.7 (0.3)	21	4.6 (0.3)	21	4.6 (0.3)	
CPHI Verbal Strategies	Control	44	2.3 (0.8)	40	2.3 (0.9)	40	2.3 (0.8)	**.03**
	Per Protocol	21	2.2 (0.5)	21	2.0 (0.4)	21	2.4 (0.7)	
CPHI Non-Verbal Strategies	Control	44	2.9 (1.0)	40	3.0 (1.0)	40	3.0 (0.9)	.45
	Per Protocol	21	3.1 (0.7)	21	2.9 (0.7)	21	3.1 (0.7)	
CPHI Self-Acceptance	Control	44	4.4 (0.7)	40	4.4 (0.6)	40	4.5 (0.5)	.54
	Per Protocol	21	4.5 (0.7)	21	4.6 (0.5)	21	4.5 (0.7)	

CPHI Stress and Withdrawal	Control	44	4.0 (0.7)	39	4.1 (0.6)	40	4.0 (0.8)	.72
	Per Protocol	21	4.1 (0.5)	21	4.2 (0.6)	21	4.1 (0.6)	
SEHHS Self efficacy for hearing help seeking	Control	43	82.1 (17.2)	38	83.2 (13.4)	39	85.7 (10.0)	.52
	Per Protocol	21	88.2 (15.3)	21	86.2 (13.5)	21	88.5 (13.4)	
AQ Benefits	Control	43	3.6 (0.5)	38	3.6 (0.4)	40	3.6 (0.5)	.07
	Per Protocol	21	3.7 (0.6)	21	3.5 (0.5)	21	3.4 (0.5)	
AQ Stigma	Control	43	2.2 (0.9)	38	2.2 (0.8)	40	2.2 (0.7)	.66
	Per Protocol	21	2.5 (0.8)	21	2.4 (0.8)	21	2.3 (0.8)	
AQ Social Pressure	Control	43	3.0 (0.9)	38	3.0 (0.9)	40	3.1 (0.9)	.08
	Per Protocol	21	3.0 (0.7)	21	2.8 (0.7)	21	2.8 (0.8)	
AQ Evaluation of Hearing Aids by Others	Control	43	2.4 (0.7)	38	2.3 (0.5)	40	2.3 (0.6)	.28
	Per Protocol	21	2.3 (0.8)	21	2.1 (0.7)	21	2.3 (0.9)	
AIADH Total	Control	44	0.7 (0.5)	40	0.6 (0.4)	41	0.7 (0.5)	.35
	Per Protocol	21	0.7 (0.3)	21	0.7 (0.3)	21	0.7 (0.4)	
AIADH Distinction of Sounds	Control	44	0.5 (0.4)	40	0.4 (0.4)	41	0.5 (0.5)	.53
	Per Protocol	21	0.3 (0.3)	21	0.4 (0.3)	21	0.5 (0.5)	
AIADH Auditory Localization	Control	44	0.7 (0.6)	40	0.6 (0.6)	41	0.7 (0.6)	.80
	Per Protocol	21	0.6 (0.5)	21	0.6 (0.5)	21	0.6 (0.5)	
AIADH Intelligibility in Noise	Control	44	1.2 (0.6)	40	1.1 (0.6)	41	1.2 (0.7)	.05
	Per Protocol	21	1.2 (0.5)	21	1.2 (0.5)	21	1.1 (0.5)	
AIADH Intelligibility in Quiet	Control	44	0.8 (0.6)	40	0.8 (0.6)	41	0.8 (0.5)	.18
	Per Protocol	21	0.9 (0.4)	21	0.8 (0.3)	21	0.8 (0.5)	
AIADH Detection of Sounds	Control	44	0.6 (0.5)	40	0.5 (0.4)	41	0.6 (0.5)	.11
	Per Protocol	21	0.5 (0.3)	21	0.6 (0.3)	21	0.5 (0.3)	

[a] p-value of the interaction between time and group.
SD = Standard deviation, LMM = Linear Mixed Model. AIADH = Amsterdam Inventory for Auditory Disability and Handicap, AQ = Attitude Questionnaire, CPHI = Communication Profile for the Hearing Impaired, PHHSS = Prior Hearing Help-Seeking Steps, PHS = Partners in Health Scale, SEHHS = Self-Efficacy for Hearing Help-Seeking Scale,
